# Lysosomal Enzyme Glucocerebrosidase Protects against Aβ_1-42_ Oligomer-Induced Neurotoxicity

**DOI:** 10.1371/journal.pone.0143854

**Published:** 2015-12-02

**Authors:** Seulah Choi, Donghoon Kim, Tae-In Kam, Seungpil Yun, Sangjune Kim, Hyejin Park, Heehong Hwang, Olga Pletnikova, Juan C. Troncoso, Valina L. Dawson, Ted M. Dawson, Han Seok Ko

**Affiliations:** 1 Neuroregeneration and Stem Cell Programs, Institute for Cell Engineering, The Johns Hopkins University School of Medicine, Baltimore, Maryland, United States of America; 2 Department of Neurology, The Johns Hopkins University School of Medicine, Baltimore, Maryland, United States of America; 3 Department of Physiology, The Johns Hopkins University School of Medicine, Baltimore, Maryland, United States of America; 4 Solomon H. Snyder Department of Neuroscience, The Johns Hopkins University School of Medicine, Baltimore, Maryland, United States of America; 5 Department of Pharmacology and Molecular Sciences, The Johns Hopkins University School of Medicine, Baltimore, Maryland, United States of America; 6 Department of Pathology, The Johns Hopkins University School of Medicine, Baltimore, Maryland, United States of America; 7 Adrienne Helis Malvin Medical Research Foundation, New Orleans, Louisiana, United States of America; 8 Diana Helis Henry Medical Research Foundation, New Orleans, Louisiana, United States of America; Foundation for Biomedical Research Academy of Athens, GREECE

## Abstract

Glucocerebrosidase (GCase) functions as a lysosomal enzyme and its mutations are known to be related to many neurodegenerative diseases, including Gaucher’s disease (GD), Parkinson’s disease (PD), and Dementia with Lewy Bodies (DLB). However, there is little information about the role of GCase in the pathogenesis of Alzheimer’s disease (AD). Here we demonstrate that GCase protein levels and enzyme activity are significantly decreased in sporadic AD. Moreover, Aβ_1–42_ oligomer treatment results in neuronal cell death that is concomitant with decreased GCase protein levels and enzyme activity, as well as impairment in lysosomal biogenesis and acidification. Importantly, overexpression of GCase promotes the lysosomal degradation of Aβ_1–42_ oligomers, restores the lysosomal impairment, and protects against the toxicity in neurons treated with Aβ_1–42_ oligomers. Our findings indicate that a deficiency of GCase could be involved in progression of AD pathology and suggest that augmentation of GCase activity may be a potential therapeutic option for the treatment of AD.

## Introduction

The abnormal deposition of aggregated proteins such as amyloid-β (Aβ), tau, α-synuclein, and TAR DNA-binding protein (TDP-43) are linked to various age-related neurodegenerative disorders [1, 2]. Alzheimer’s disease (AD) is the most common age-related neurodegenerative disorder, the pathological hallmark of which is the accumulation of oligomeric Aβ peptides and tau species that lead to neuritic plaques and neurofibrillary tangles. Growing evidence suggests that early stages of AD are driven by the oligomerization of Aβ, which plays a critical role in the formation of Aβ deposits in patients with AD [3].

One of the most recognized risk factors of AD is aging. As humans age, degradation mechanisms lose efficiency, resulting in aberrant aggregate levels of numerous types of cellular proteins. It is well documented that diminished proteasomal and autophagic-lysosomal activity may be a major contributor to AD pathology, since malfunctioning degradation systems lead to Aβ peptide accumulation. This may increase the selective vulnerability of neurons and could ultimately have a substantial impact on the disease process [3–6]. The autophagic-lysosomal system is one of the main cellular pathways that promote Aβ degradation [7, 8]. Therefore, recent therapeutic strategies have focused on enhancing autophagy-lysosome-mediated degradation of Aβ as a treatment for AD [9, 10].

Glucocerebrosidase (GCase) is a lysosomal enzyme that catalyzes the breakdown of glucosylceramide to glucose and ceramide. *GBA1* deficient mutants have been associated with several neurodegenerative disorders. Homozygous mutations in the *GBA1* gene are associated with Gaucher’s disease (GD), a lysosomal storage disorder [11, 12]. In addition, heterozygous mutations in *GBA1* are significant genetic risk factors in the progression of Parkinson’s disease (PD) [13–15] and Dementia with Lewy bodies (DLB) [16–18]. While the precise molecular mechanisms by which *GBA1* mutations contribute to these diseases are still not known, much evidence indicates that *GBA1* mutations in selectively vulnerable neurons lead to a reduction in GCase protein levels and activity. Accompanying these changes in GCase in PD and DLB is the accumulation of α-synuclein and glucosylceramide, as well as autophagic-lysosomal defects [19–22]. GCase protein levels and activity are considerably lower in the substantia nigra of the PD post-mortem brain even without the *GBA1* mutations [22–24], and the accumulation of α-synuclein leads to a substantial reduction of GCase levels and activity [19, 22]. Additionally, the GCase deficit influences the rate of cell-to-cell spreading of α-synuclein amyloids, suggesting that the lysosomal function of GCase may be a key modulator of the disease process [25]. On the other hand, the enhancement of GCase activity via gene therapy or pharmacological chaperones that target GCase yields beneficial effects on the neurodegeneration phenotype observed in PD and α-synucleinopathy *in vivo* [26, 27]. However, there is little information about GCase deficiency and its potential contribution to AD pathogenesis. Given that lysosomal function is also critical to disease development of AD and is involved in the accumulation of Aβ, it is reasonable to presume that GCase overexpression may lead to enhancement of GCase activity that could ameliorate the disease process.

To investigate the potential relationship between AD pathology and lysosomal dysfunction caused by GCase deficiency, we sought to explore whether there are aberrant GCase protein levels and activity in brains affected by AD. Moreover, we assessed whether the enhancement of GCase levels and activity could accelerate the lysosomal degradation of Aβ_1–42_ oligomers and ameliorate Aβ_1–42_ oligomer-induced neuronal toxicity. In this study, we demonstrate that there was a substantial decrease in GCase protein levels and enzyme activity in post-mortem hippocampal brain tissue of AD patients and Aβ_1–42_ oligomer-treated primary neurons. We also found that augmentation through the ectopic expression of GCase protects against Aβ_1-42_-induced neuronal toxicity.

## Materials and Methods

### Ethics statement

For use of human post-mortem brain tissues in this research, patients provided written informed consent and approval for the consent procedure and research were obtained from the Johns Hopkins Institutional Review Boards (Approval No. NA00032761). Human post-mortem brain tissues were obtained through the brain donation program of the Alzheimer's Disease Research Center (http://www.alzresearch.org) and the Morris K. Udall Parkinson's Disease Research Center of Excellence at Johns Hopkins Medical Institutions (JHMI) in compliance with local Institutional Review Board and HIPAA (Health Insurance Portability and Accountability Act) regulations.

### Preparation of synthetic Aβ_1–42_ oligomers

The synthetic Aβ_1–42_ peptide was purchased from rPeptide (Cat#: A-1163-2). 2.2 mM Aβ_1–42_ peptides solution was obtained by dissolving the peptides in DMSO, and then further diluted in PBS to obtain a 250 μM stock solution. Cross-linking of the peptides occurred while the solution was incubated at 4°C for at least 24 hours. After cross-linking, the solution was aliquoted and stored at -80°C until use. Before usage, the solution was centrifuged at 12,000 X g for 10 minutes to remove the fibril forms of Aβ_1–42_, which would precipitate. Then, the dissolved oligomeric Aβ_1–42_ that was present in the supernatant was used for experiments. The Aβ_1–42_ preparation was confirmed by immunoblot using anti-Aβ antibody (Covance, clone: 4G8) [28].

### Primary neuronal cultures

Hippocampal and cortical neurons were prepared from embryonic day 15 CD-1 mice (Charles River, Wilmington, MA), as previously described [28, 29]. Dissociated neurons were plated onto dishes coated with poly-D-lysine (Sigma-Aldrich) while submerged in the culture medium that consisted of Neurobasal Media (Invitrogen), containing B27 supplement and L-glutamine (Gibco). The medium was changed twice a week and the cultures were maintained in 7% CO_2_ incubator at 37°C. 5 days after the culture, 30 μM 5-fluoro-2’-deoxyuridine was added to the cultures to inhibit glial cell growth. All procedures involving mice were approved by and conformed to the guidelines of the Johns Hopkins University Animal Care and Use Committee.

### Production of lenti-viral vector and virus, and treatment of lenti-virus

For preparation of GCase lenti-virus, human complementary DNA of *GBA1* was subcloned into a lenti-viral cFUGW vector by enzyme Age I, and the positive clone was sequenced. Lenti-viruses were prepared as previously described [30]. Briefly, the cFUGW-*GBA1* plasmid or cFUGW plasmid was transiently transfected into HEK293FT along with viral packaging plasmids purchased from Invitrogen using lipofectamine LTX with plus transfection reagent. Infectious lenti-viruses were harvested at 48 hours post-transfection. The supernatant was collected, filtered, concentrated by ultracentrifugation at 25,000 X g for 3 hours, re-suspended in 1% BSA in PBS, and then stored at -80°C until use. Viral particle content was assayed for the p24 core antigen by using the p24 antigen enzyme-linked immunosorbent assay (RETROtek, 22-157-319) according to the manufacturer’s instructions. Titer was measured using the Lenti-X™ qRT-PCR titration kit (Clontech) according to the manufacturer’s instructions. Lenti-cFUGW-GCase or lenti-cFUGW virus was used to overexpress GCase or control with a multiplicity of infection (MOI) of 5 in primary neurons at 7 days *in vitro*.

### Immunoblot analysis

Human hippocampal post-mortem tissues or mouse cortical neurons were homogenized and prepared in lysis buffer {for tissues, 10 mM Tris-HCl, pH 7.4, 150 mM NaCl, 5 mM EDTA, 0.5% Nonidet P-40, 10 mM Na-β-glycerophosphate, phosphatase inhibitor cocktail I and II (Sigma-Aldrich), and complete protease inhibitor cocktail (Roche); for mouse cortical neurons, 50 mM Tris-HCl, 150 mM NaCl, 1% NP-40, 0.5% sodiumdeoxychrolate, 1 mM EDTA, 1% SDS, and protease inhibitor as previously described [31, 32]. For subcellular fractionation of primary cultured neurons, the buffer containing 0.25 M sucrose, 10 mM HEPES (pH 7.4) and 0.1 M EDTA was used to lyse the cells. The lysate is then homogenized and centrifuged at 6,800 X g, 4°C, for 5 minutes. The supernatant was further centrifuged at 17,000 X g for 10 min, and the pellet enriched with lysosomes was harvested in the lysis buffer described above. 2X Laemmli buffer (Bio-Rad) was utilized to dilute the lysates. Electrophoresis on 8–16% and 4–20% gradient SDS-PAGE gels (Life technologies) was performed in order to resolve proteins from the human hippocampal post-mortem tissue (20 μg) and proteins from the mouse cortical neuron (10 μg) respectively. The proteins were then transferred to nitrocellulose membranes. The membranes were blocked with Tris-buffered saline (5% non-fat dry milk and 0.1% Tween-20) for 1 hour and incubated at 4°C overnight with primary antibodies: anti-GCase (1:1000, G4171, Sigma), anti-Amyloid (1:1000, clone: 4G8, BioLegend), anti-cathepsin D (1:2000, ab6313, Abcam), MEK1/2 (1:1000, #8727, Cell Signaling Technology) or anti-LAMP1 (1:1000, ab24170, Abcam) antibodies, followed by HRP-conjugated rabbit or mouse secondary antibodies (GE Healthcare) for 1 hour at room temperature. Chemiluminescence (Thermo Scientific) was utilized in order to visualize the immunoblot signals. The membranes were re-probed with HRP-conjugated β-actin antibody (1:30,000, Sigma). For lysosome enzyme inhibition, E-64d and Pepstatin A (PepA) were purchased from Sigma-Aldrich.

### Glucocerebrosidase activity assay

The GCase activity assay has been performed as described [19, 33]. Human hippocampal post-mortem tissues and primary cultured neuron samples were gathered in the buffer containing 0.25 M sucrose, 10 mM HEPES (pH 7.4) and 0.1 M EDTA, and the samples were homogenized and centrifuged at 6,800 X g, 4°C, for 5 minutes. The supernatant was then centrifuged at 17,000 X g for 10 min, and the pellet enriched with lysosomes was harvested in 50 μl of activity assay buffer (0.25% Triton X-100 (Sigma-Aldrich), 0.25% Taurocholic acid (Sigma-Aldrich), 1 mM EDTA, in citrate/phosphate buffer, pH 5.4). The samples were then frozen and thawed twice, and iced for 30 minutes. 10 μl of supernatant was obtained after the samples were centrifuged at 20,000 X g for 20 minutes, which was then used to measure GCase activity in 50 μl of 1% BSA, with 1 mM 4-Methylumbelliferyl β-glucophyranoside (4-MU; M3633, Sigma-Aldrich) and/or 10 mM conduritol B epoxide (CBE, Sigma-Aldrich). The reaction was further incubated for 40 minutes at 37°C, and 50 μl (equi-volume) of 1M glycine at pH of 12.5 was added to halt the reaction. 100 μl samples were prepared for fluorescence testing (Nunc, # 136101), which was measured via a Perkin Elmer plate reader (ex = 355 nm, em = 460 nm, 0.1 s). GCase1 activity was obtained by subtracting the GCase activity in presence of CBE from the total GCase activity of each sample. 95–97% of GCase activities were reduced by CBE treatment. Fluorescence intensity was converted to actual enzyme activities using a 4-MU standard curve by human recombinant GCase (7410-GH, R&D systems).

### β-galactosidase activity assay

The pellet enriched with lysosomes was harvested as described above in Glucocerebrosidase activity assay. β-galactosidase activity in lysosome-enriched fraction was determined with the β-Gal Assay kit (Life technologies, #K1455-01) according to the manufacturer’s instructions.

### Endoglycosidase H resistant assay

As previously described [19], Triton-X100 soluble lysates (25 μg) were denatured in 10 μl of Glycoprotein Denaturing Buffer and digested with 500 U of Endo H (New England Biolabs) for 1 hour at 37°C. Control reactions were incubated in the same condition without Endo H enzyme.

### Cell viability analysis

Cell viability was tested via two methods; The DeadEnd^TM^ Fluorometric TUNEL system (Promega) and LDH assay (Sigma). Cell death was assessed through the TUNEL assay, according to the manufacturer’s protocol. Nuclei were stained with Hoechst (Life technologies). LDH activity in culture medium, representing relative cell viability and membrane integrity, was measured by spectrophotometer using the LDH assay kit, following the manufacturer’s instructions.

### Real-time PCR

Using RNeasy® Plus Micro Kit (Qiagen), total RNA contents were isolated from human hippocampal post-mortem tissues. SuperScript® IV First-Strand Synthesis System (Life technologies) was used to synthesize the first-stand cDNA. Real-time PCR was conducted with the SYBR Green reagent and a ViiA™ 7 real-time PCR system (Life technologies). According to the 2^−ΔΔCT^ method [34], all ΔC_T_ values were normalized to β-actin. The primer sequences used for real-time PCR were as flows: human *β-actin* forward, 5’-ATT GCC GAC AGG ATG CAG AAG-3’; human *β-actin* reverse, 5’-TTG CTG ATC CAC ATC TGC TGG-3’; human *GBA1* forward, 5’-CAG CCT CAC AGG ATT GCT TCT-3’; human *GBA1* reverse, 5’-GAC ACA CAC CAC CGA GCT GTA-3’.

### Immunofluorescence

Coverslips with poly-D-lysine coating were utilized in order to plate the primary mouse cortical neurons at a concentration of 20,000 cells/cm^2^. 4% paraformaldehyde was used to fix the neurons, followed by blocking in a solution with 5% normal donkey serum (Jackson ImmunoResearch, BarHarbor, ME), 2% BSA (Sigma) and 0.1% Triton X-100 (Sigma) for 1 hour at room temperature. A series of incubations with anti-LAMP1 (1:1,000, Abcam) and anti-cathepsin D (1:1,000, Abcam) antibodies or anti-LAMP1 (1:1,000, Abcam) and anti-MAP2 (1:1000, MAB3418, Millipore) antibodies followed at 4°C overnight. The samples were washed with 0.1% Triton X-100 in PBS, followed by 1 hour of incubation of the coverslips with a mixture of FITC-conjugated (Jackson Immunoreserach) and Cy3-conjugated (Jackson Immunoreserach) secondary antibodies at room temperature. The fluorescent images were acquired via a Zeiss confocal microscope (Zeiss Confocal LSM 710) after the coverslips were mounted on microscope slides

### Quantification of lysosomal area and size

Primary mouse cortical neurons were plated onto 18 mm coverslips coated with poly-D-lysine in a 12-well plate (1 × 10^5^ cells/coverslip). After 7 days *in vitro*, neurons were infected with control or GCase lenti-viral vector (MOI = 5) for 72 hours and then incubated with 1 μM oligomeric Aβ_1–42_ and CellLight® plasma membrane-CFP (C10606; Life technologies) for 24 hours. For measuring of lysosomal area and size, the lysosomes were stained with Lysotracker Red DND-99 (L7528; Life technologies) following the manufacturer’s instructions. The areas of positive-Lysotracker and diameters of puncta were measured using ImageJ software in at least 15 randomly selected non-overlapping images per each group. Each image contained 3–4 cells.

### Cytosolic pH measurement

For intracellular pH measurement, primary cortical neurons were incubated with 10 μM of pHrodo™ Green and Red AM intracellular pH indicators for 30 minutes at 37°C according to the manufacturer’s protocol (P35380; Life technologies). Standard curves of cytosolic pH were created using pHrodo™ Green and Red AM with intracellular pH calibration buffer kit (P35379; Life technologies) that contains a pH range of (pH 4.5, 5.5, 6.5, and 7.5), as well as valinomycin and nigericin, which helps equilibrate the pH inside and outside of cells. The fluorescence images were obtained using a confocal microscope (Zeiss Confocal LSM 710) and the intensities were analyzed using ImageJ (NIH, http://rsb.info.nih.gov/ij/).

### Cathepsin D activity assay

Lysosome-enriched fraction and cytoplasmic fraction were prepared as described above in glucocerebrosidase activity assay. The lysosome-enriched fraction was obtained from the pellet after centrifugation at 17,000 X g for 10 minutes, and the supernatant was used as the cytoplasmic fraction. Equal amounts of protein (15 μg) were used for the cathepsin D activity assay. Fluorescence values of these samples were measured using cathepsin D Activity Assay Kit/Fluorometric (Abcam, ab65302), following the manufacturer’s protocol. The actual enzyme activities were then calculated using a standard curve of the substrate by cathepsin D (C3138, Sigma).

### Statistics

Data were presented as mean ± SEM from at least 3 independent experiments. In order to assess statistical significance, Student’s t tests or ANOVA tests followed by Bonferroni post hoc analysis were performed using GraphPad Prism software. Assessments with a p <0.05 were considered significant.

## Results

### GCase protein levels and enzyme activity are decreased in sporadic AD and Aβ_1–42_ oligomer-treated neurons

**Fig 1:**
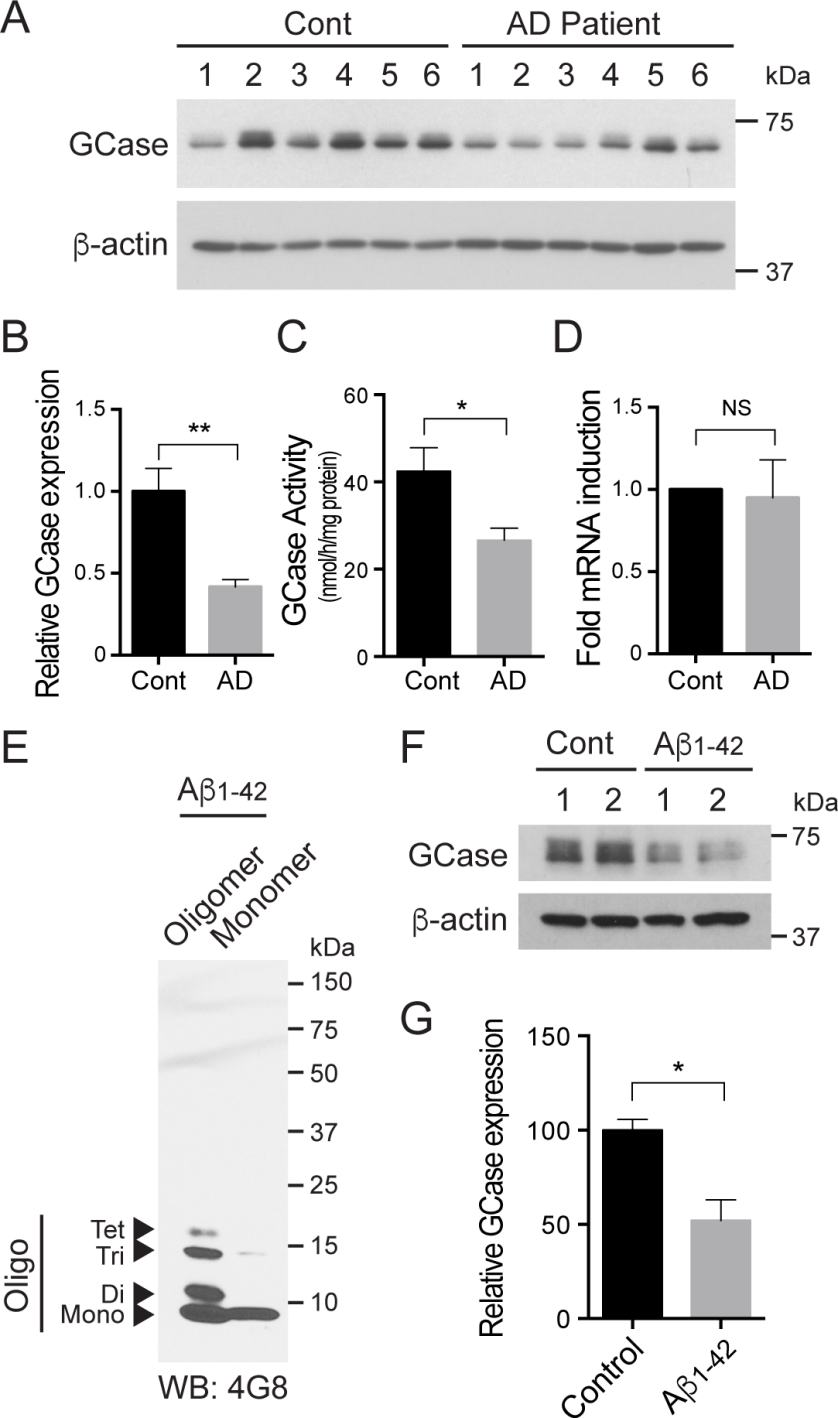
GCase protein levels and activity are reduced in sporadic AD brain tissues and Aβ_1–42_ oligomer-treated primary neurons. (A and B) GCase expression was decreased in the hippocampal region of brains affected by Alzheimer’s disease (n = 6), as opposed to controls (n = 6). GCase expression levels were normalized to β-actin and quantified. (C) The GCase enzymatic activities were measured using lysosome-enriched fractions for the brains affected by Alzheimer’s disease and the controls (n = 6 per group). (D) Expression of *GBA1* gene was determined in AD hippocampal regions compared to the controls using real-time PCR. The β-actin mRNA was used as an internal reference control to normalize relative mRNA levels (n = 6 per group). (E) Oligomeric and monomeric Aβ_1–42_ were identified by SDS-PAGE using anti-Aβ antibody (4G8). (F) Primary cortical neurons were incubated with 1 μM oligomeric Aβ_1–42_ for 24 h at 10 days *in vitro*. The cell lysates were subjected to immunoblot using GCase and β-actin antibodies. (G) The expression level of GCase was quantified. Data are expressed as the mean ± SEM (Student’s t test, *P < 0.05, **P < 0.01).

**Fig 2:**
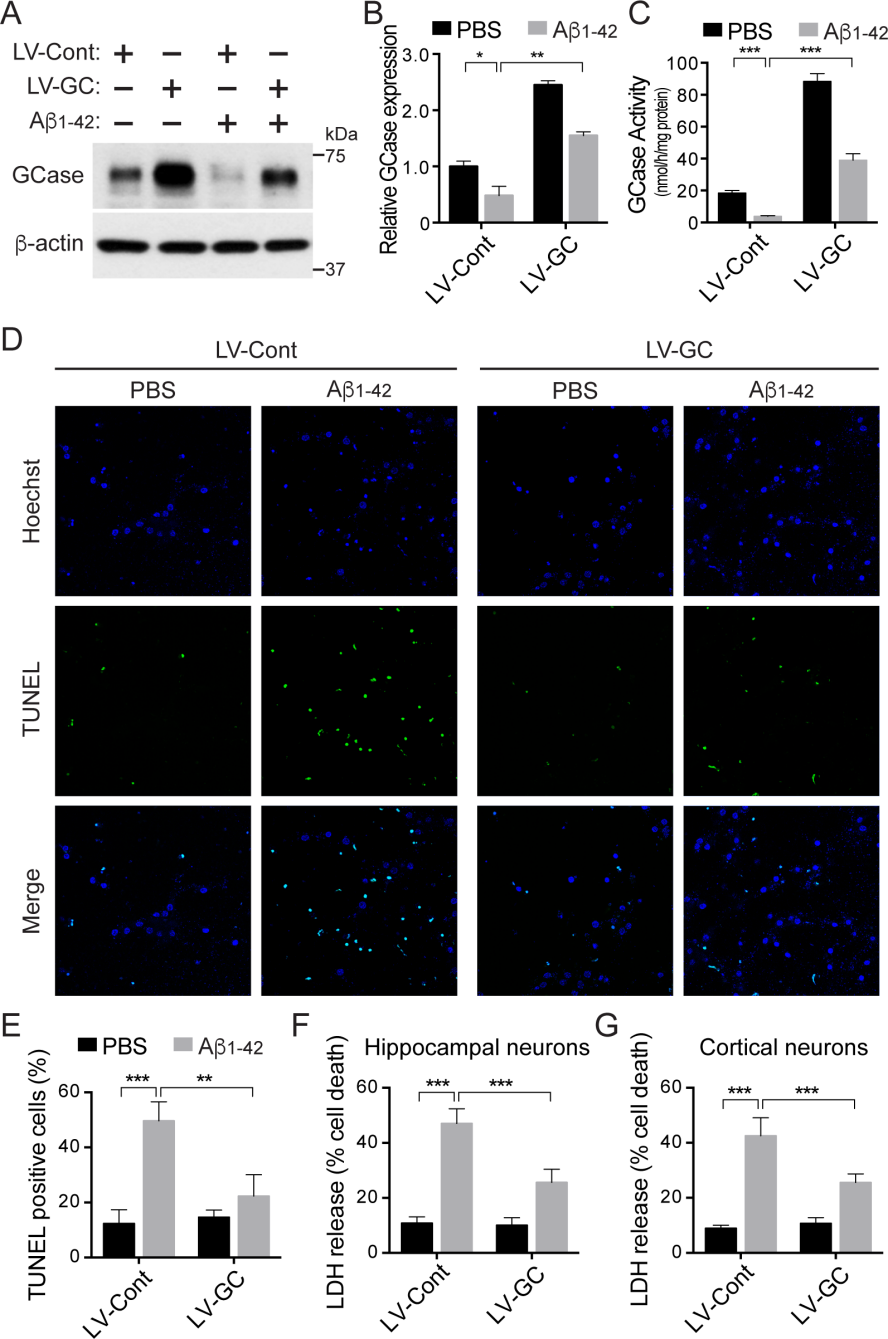
GCase protects against Aβ_1–42_ oligomer-induced neuronal cell death. (A, B and C) Primary mouse hippocampal neurons expressing lenti-control (LV-Cont, n = 3–4 per group) or lenti-GCase (LV-GC, n = 3–4 per group) were treated with 5 μM oligomeric Aβ_1–42_ or PBS for 48 h at 10 days *in vitro*, and subjected to immunoblot analysis with anti-GCase antibody and to GCase enzyme activity assay. (D and E) The neuronal cell death was determined by the quantification of TUNEL positive-cells (n = 3–4 per group). Primary mouse hippocampal (F) or cortical (G) neurons were infected with lenti-control or lenti-GCase at 7 days *in vitro*, and then further incubated with 5 μM oligomeric Aβ_1–42_ or PBS for 48 h at 10 days *in vitro*. The cell death was assessed via LDH assay. Data represent the mean ± SEM (two-way ANOVA, Bonferroni posttest, P* < 0.05, P** < 0.01, P*** < 0.001, n = 5 for hippocampal neurons, n = 6 for cortical neurons).

To evaluate the potential relationship between GCase deficiency and AD pathogenesis, we monitored GCase protein levels and enzyme activity in human post-mortem tissue of AD patients and age-matched controls (S1 Table). Whole tissue lysates were prepared from human AD post-mortem hippocampus brain tissues, followed by immunoblot analysis. In hippocampal AD patient samples, there is a 58% reduction in GCase protein levels when compared to the controls (Fig 1A and 1B). Accompanying the reduction of GCase proteins, there is also a 37% reduction of GCase enzyme activity in hippocampal lysosome-enriched samples from AD patients over the age-matched controls (Fig 1C). Interestingly, the activity of other lysosomal hydrolase enzymes, such as β-galactosidase, is also reduced in AD patients’ brain samples when compared to the controls (S1A Fig). To verify whether alterations in GCase glycosylation patterns lead to lower protein levels and lysosomal activity of GCase seen in AD post-mortem samples, the levels of mature lysosomal GCase were analyzed by Endo H treatment. This analysis reveals no change in the levels of Endo H-resistant GCase in AD hippocampus compared to the control hippocampus, indicting that the lowered protein levels and lysosomal GCase function in AD are unlikely due to an abnormal trafficking of GCase (S1B Fig). To ascertain whether the reduction of GCase is caused by the alteration in *GBA1* mRNA levels, real-time PCR was performed. This reveals no difference in the mRNA levels of GCase between the AD and control samples (Fig 1D), indicating that the reduction of GCase protein levels and activity is possibly due to their abnormal protein turn over. Given that Aβ_1–42_ oligomer treatment recapitulates the phenotype found in AD [35], we asked whether the treatment of Aβ_1–42_ oligomers could lead to decrements of GCase protein levels and enzyme activity. Oligomeric species of Aβ_1–42_ were prepared, and the toxic oligomeric species and non-fibril particles were confirmed by immunoblot analysis (Fig 1E) [28]. To ascertain whether the prepared Aβ_1–42_ oligomers could lead to decreased GCase protein levels and activity, we treated primary cortical neurons with Aβ_1–42_ oligomers (1 μM) and performed an immunoblot analysis. Consistent with the results from the human AD post-mortem hippocampus brain tissue, there are a 48% reduction in GCase protein levels (Fig 1F and 1G) and a 79% decrease in GCase enzyme activity (Fig 2C). Taken together, these data indicate that GCase deficiency as indicated by reduced GCase protein expression and enzyme activity, in part, may be a contributor to the progression of AD pathogenesis.

### GCase protects against Aβ_1–42_ oligomer-induced neuronal cell death

Our findings of reduced GCase protein levels and activity in AD brain samples and Aβ_1–42_ oligomer-treated primary neurons prompted us to examine the potential neuroprotective role of GCase in Aβ_1–42_ oligomer-induced toxicity. Primary hippocampal or cortical neurons at 7 days *in vitro* were infected with either lenti-control or lenti-GCase virus, and treated with Aβ_1–42_ oligomers at 10 days *in vitro* for 48 hours. Overexpression of GCase leads to a 245% increase in GCase protein levels and a 480% increase in GCase activity (Fig 2A, 2B and 2C). Neuronal death in response to Aβ_1–42_ toxicity was monitored through TUNEL staining and LDH measurement. The overexpression of GCase significantly reduces the Aβ_1–42_ oligomer-induced toxicity in primary hippocampal neurons by 80% and 57% when observed through the TUNEL staining and the LDH assay, respectively (Fig 2D, 2E and 2F). Similar results were observed in primary cortical neurons through the LDH assay (Fig 2G). These data indicate that increased GCase expression rescues Aβ_1–42_ oligomer-induced neuronal cell death.

### GCase accelerates Aβ_1–42_ oligomer degradation

**Fig 3:**
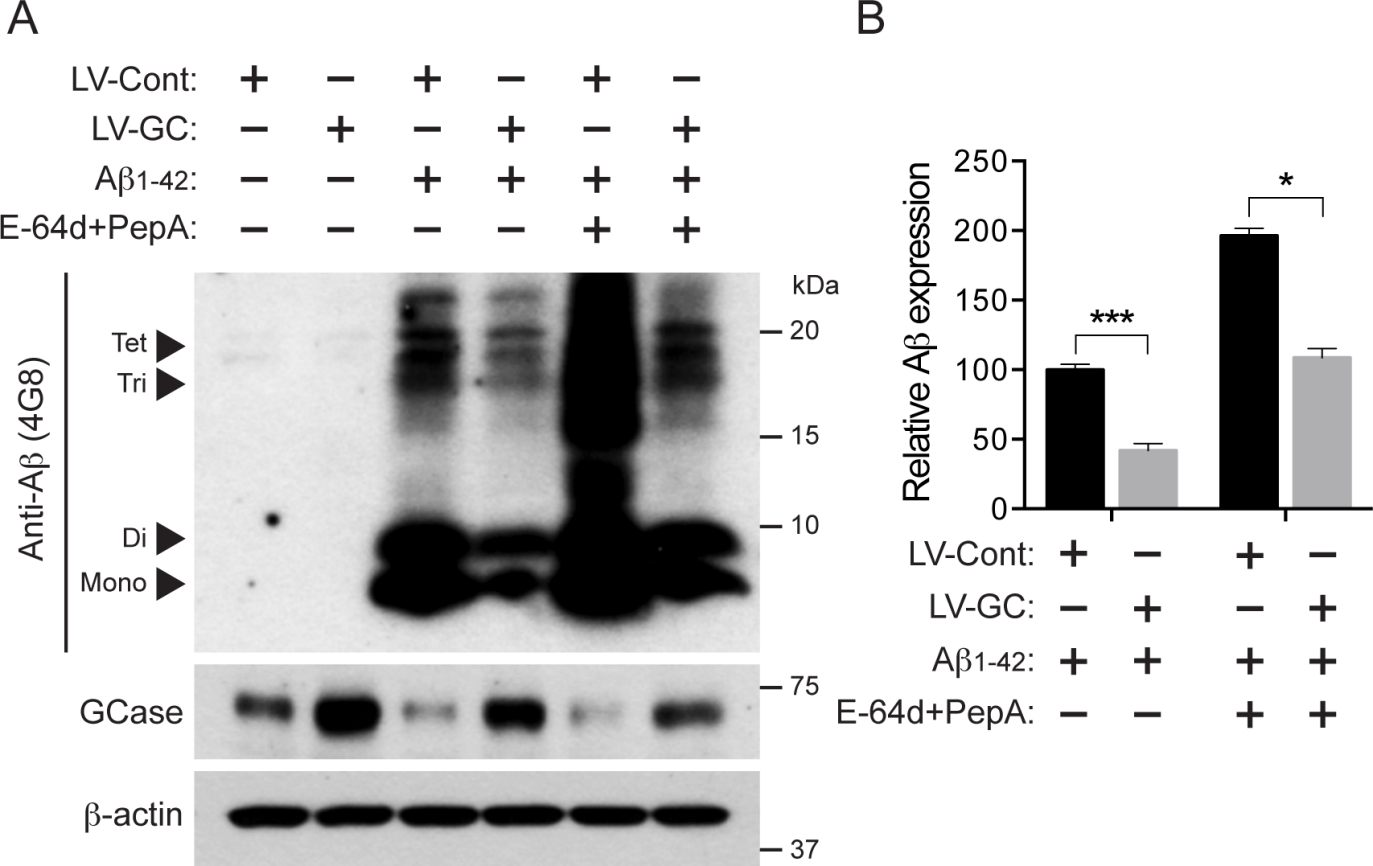
GCase promotes Aβ_1–42_ oligomer degradation. (A) Primary cultured mouse cortical neurons were infected with lenti-control (LV-Cont, n = 3) or lenti-GCase (LV-GC, n = 3) virus at 7 days *in vitro*, and 5 μM oligomeric Aβ_1–42_ or PBS was added for 48 h at 10 days *in vitro*. 10 μg/ml of E-64d and 10 μg/ml of PepA were treated 4 h before the treatment of Aβ_1–42_ oligomers. The monomeric and oligomeric Aβ expressions were measured with anti-Aβ antibody (4G8) 24 h after the treatment of oligomeric Aβ_1–42._ (B) The quantification of Aβ levels is shown (Student’s t test, P* < 0.05, P*** < 0.001).

Since GCase localizes in the lysosome compartment and Aβ oligomers are degraded through the lysosomal degradation pathway [7, 8, 36], we investigated whether GCase overexpression can regulate Aβ oligomer degradation. Primary cortical neurons were infected with either lenti-control or lenti-GCase virus, and subjected to Aβ_1–42_ treatment. Aβ immunoblot analysis revealed that ectopic expression of GCase dramatically decreases Aβ_1–42_ oligomer levels, indicating that overexpression of GCase promotes intra-lysosomal degradation of Aβ_1–42_ oligomers by increasing lysosomal GCase activity (Fig 3A and 3B). In addition, there are approximately two- to three-fold increased levels of Aβ_1–42_ oligomers when treated with the lysosomal enzyme inhibitors, E-64d+PepA. This indicates that the clearance of Aβ_1–42_ oligomers is regulated by the autophagic-lysosomal system. These accumulations of Aβ_1–42_ oligomers are again reduced by the overexpression of GCase (Fig 3A and 3B). These data suggest that GCase regulates Aβ_1–42_ oligomer levels, which potentially protects against Aβ_1–42_ oligomer-induced neuronal toxicity.

### GCase alleviates Aβ_1–42_ oligomer-induced lysosomal impairment

**Fig 4:**
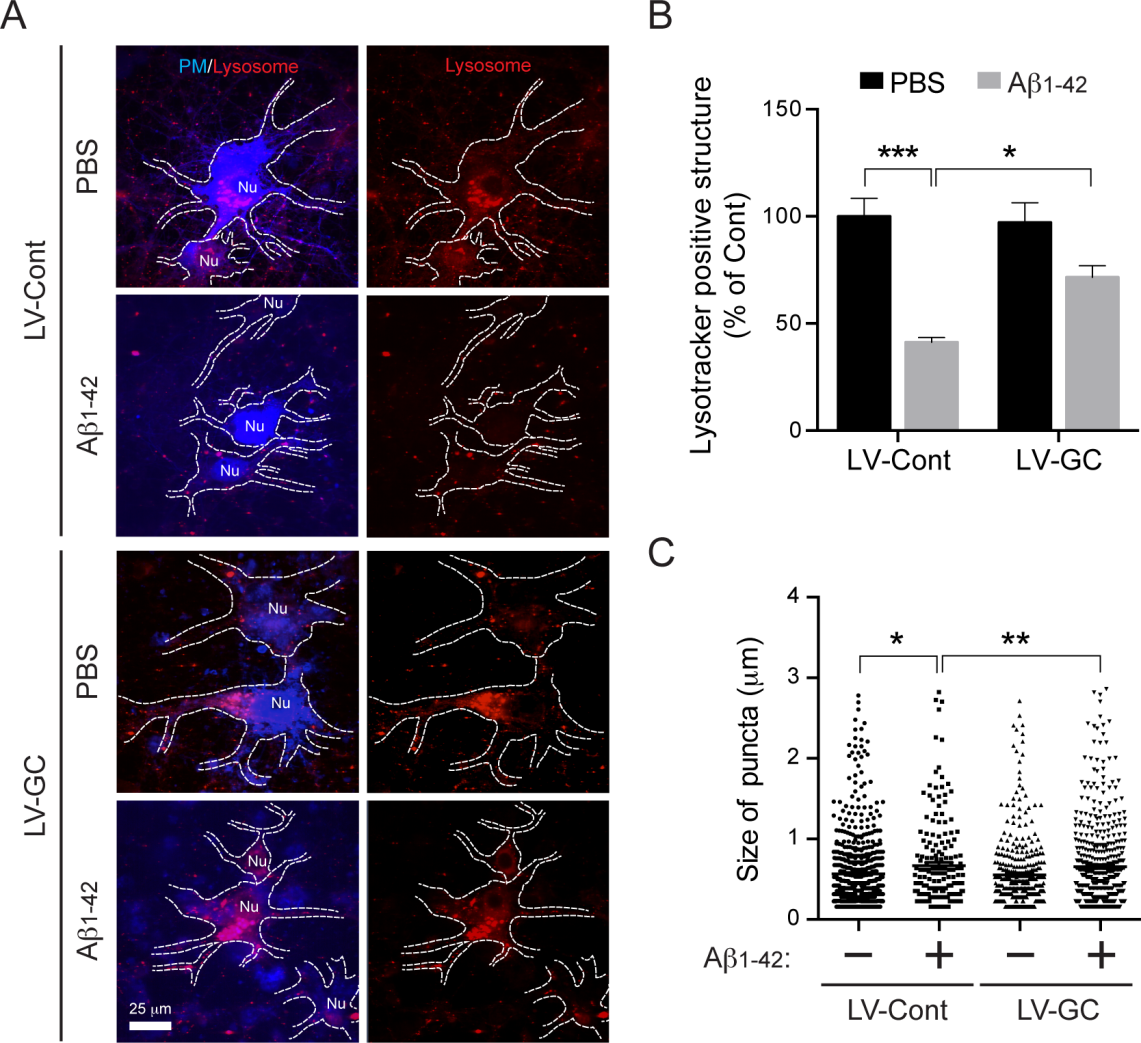
Overexpression of GCase restores impairment of lysosomal biogenesis induced by Aβ_1–42_ oligomers in neurons. (A) Primary mouse cortical neurons were infected with lenti-control (LV-Cont, n = 3) or lenti-GCase (LV-GC, n = 3) virus at 7 days *in vitro*. 72 h after the infection, the cells were further incubated with PBS or 1 μM oligomeric Aβ_1–42_ for 24 h. The plasma membranes were labeled with CellLight plasma membrane-CFP (PM, blue) and the lysosomes were labeled with LysoTracker Red DND-99 (Lysosome, red). (B) The relative area of lysosomes in plasma membrane (dotted outline) was measured (two-way ANOVA, Bonferroni posttest, P* < 0.05, P*** < 0.001). (C) The diameters of LysoTracker positive-puncta were quantified and represented as a scatter plot (two-way ANOVA, Bonferroni posttest, *P < 0.05, **P < 0.01) versus control (LV-Cont, PBS).

Aβ_1–42_ oligomer-induced neurotoxicity is due to lysosomal dysfunctions involving impaired lysosomal biogenesis, acidification and reduced cathepsin D activity in the lysosome [37, 38]. Therefore, we investigated whether overexpression of GCase restores these Aβ_1–42_ oligomer-induced lysosomal dysfunctions. Using imageJ software, lysosomal area was assessed via quantifying the LysoTracker positive puncta, and the lysosomal size was calculated via the diameter of the LysoTracker positive puncta area within each cell. LysoTracker positive puncta area per cell is reduced by 59% following treatment of cortical neurons with Aβ_1–42_ oligomers. Notably, GCase overexpression restores lysosomal area to 72% of the control neurons (Fig 4A and 4B). The reduced lysosome size observed in primary cortical neurons treated with Aβ_1–42_ oligomers is also restored by overexpression of GCase (Fig 4C). Similar results in lysosomal sizes and structures shown in Fig 4A using LysoTracker are observed in the primary neurons stained with a lysosome marker, LAMP1 (S2 Fig).

**Fig 5:**
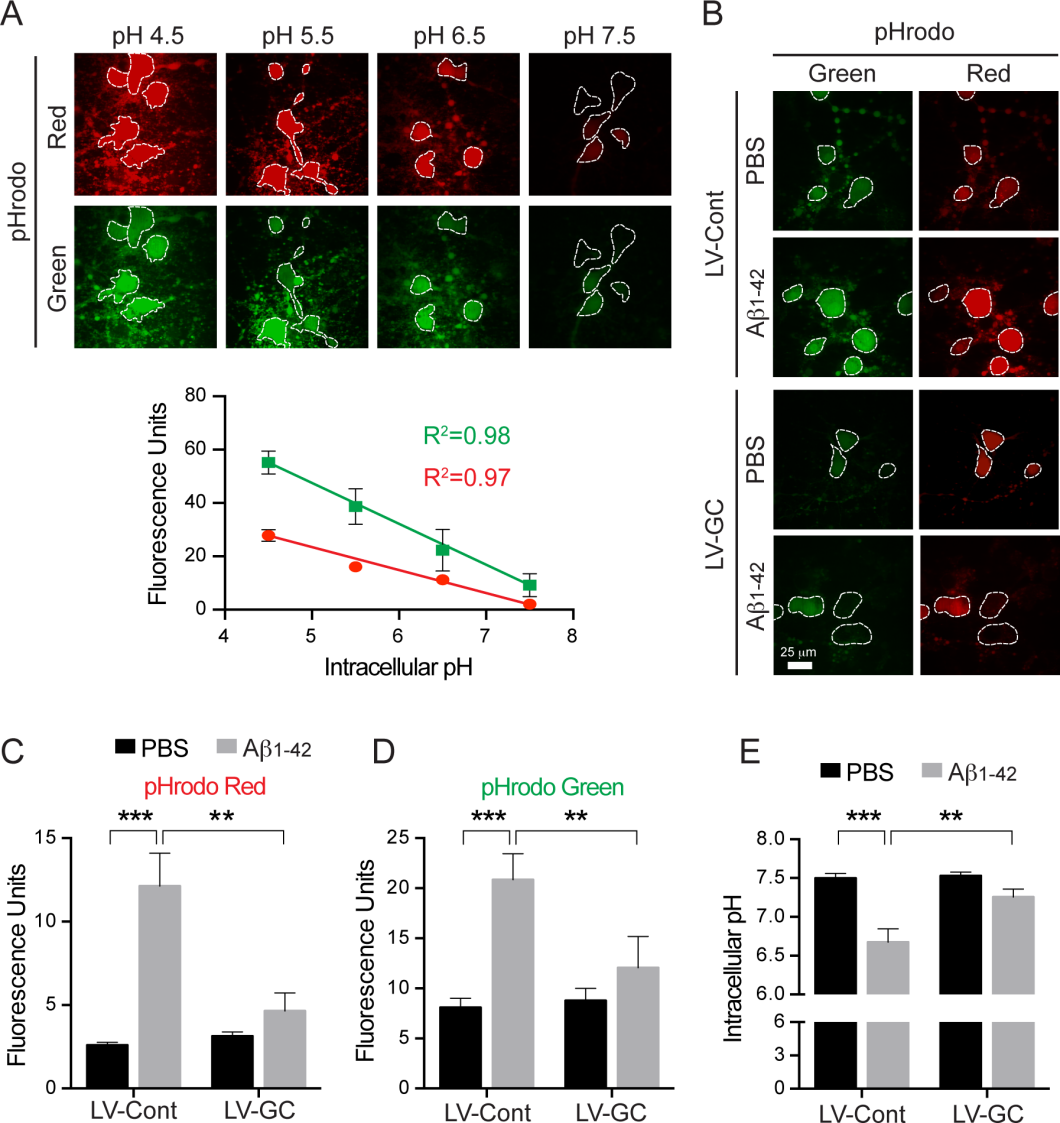
Overexpression of GCase increases intracellular pH levels reduced by Aβ_1–42_ oligomers in primary neurons. (A) Primary mouse cortical neurons were incubated with pHrodo™ Red or pHrodo™ Green at 37°C for 30 minutes. The fluorescence intensities in the intracellular area (dotted outline) were measured using various pH ranges (4.5, 5.5, 6.5, and 7.5) of intracellular pH calibration buffers. The fluorescence units of pHrodo™ Red and pHrodo™ Green signals are represented as a standard curve. (B) Primary mouse cortical neurons infected with lenti-control (LV-Cont, n = 3) or lenti-GCase (LV-GC, n = 3) virus at 7 days *in vitro* were incubated with PBS or 1 μM oligomeric Aβ_1–42_ for 24 h at 10 days *in vitro*. The neurons were loaded with pHrodo™ Red and pHrodo™ Green and live cell imaging solution, and further incubated at 37°C for 30 minutes. (C and D) The fluorescence units were quantified and represented as a graph. (E) The fluorescence units of pHrodo™ Green were converted to pH and represented as a graph (two-way ANOVA, Bonferroni posttest, **P < 0.01, ***P < 0.001).

Since several studies suggest that lysosomal dysfunction leads to decreased cytosolic pH levels [39, 40], we asked whether Aβ_1–42_ oligomer treatment influences the acidification of the cytosol in cortical neurons. Assessment of cytosolic pH was determined by the intensity of fluorescence of pHrodo Red or pHrodo Green AM fluorogenic dye in cortical neurons treated with Aβ_1–42_ oligomers or PBS. We observed that the fluorescence of both indicators increases as pH decreases (Fig 5A). Aβ_1–42_ oligomer treatment lowers cytosolic pH from pH 7.5 ± 0.1 to 6.7 ± 0.2 (Fig 5E), suggesting that lysosomal dysfunction is present. Remarkably, overexpression of GCase restores the lowered cytosolic pH induced by Aβ_1–42_ oligomer treatment to pH 7.2 ± 0.1 in cortical neurons (Fig 5B, 5C, 5D and 5E). Taken together, these findings indicate that the protective effect of GCase overexpression may be based on the restoration of impaired lysosomal function induced by Aβ_1–42_ oligomers.

**Fig 6:**
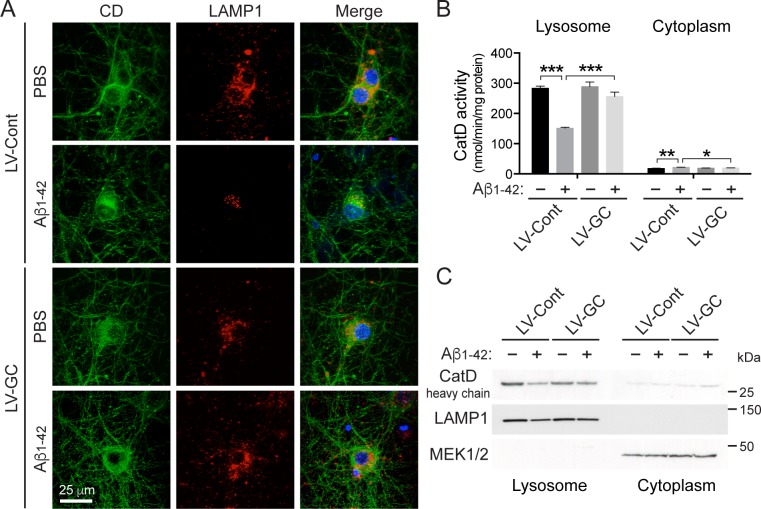
Ectopic expression of GCase restores lysosomal cathepsin D activity reduced by Aβ_1–42_ oligomer treatment in primary neurons. (A) The localization of cathepsin D is represented using lysosomal marker (LAMP1) and DAPI. (B) The cathepsin D activity levels were measured in lysosome-enriched and cytoplasmic fractions of lenti-control (LV-Cont, n = 3) or lenti-GCase (LV-GC, n = 3) virus infected primary mouse cortical neurons treated with PBS or 1 μM oligomeric Aβ_1–42_ for 24 h at 10 days *in vitro*. (C) Immunoblot analysis for heavy chain subunit of mature cathepsin D (CatD heavy chain) in cytoplasmic and lysosome-enriched fractions was performed using anti-cathepsin D antibody. The quality of cytosolic and lysosome-enriched fractions was confirmed with immunoblotting using anti-MEK1/2 and LAMP1 antibodies, respectively. The values are the mean ± SEM of three independent experiments (two-way ANOVA, Bonferroni posttest, *P < 0.05, **P < 0.01, ***P < 0.001).

Next, we monitored the lysosomal activity of cathepsin D, a main lysosomal enzyme. Consistent with previous studies [37, 38], there is a change in the localization of cathepsin D from the lysosome to the cytosol upon treatment with Aβ_1–42_ oligomers. In Aβ_1–42_ oligomer-treated neurons, there is reduced overlap of cathepsin D and LAMP1, suggesting a decrease in the level of lysosomal cathepsin D. When GCase levels were increased, the overlap of cathepsin D and LAMP1 increases to levels similar to those seen in the control (Fig 6A). Moreover, the cathepsin D activity in the lysosomal fraction prepared from primary neurons treated with Aβ_1–42_ oligomers is reduced by 47%. Notably, overexpression of GCase restores the reduction of lysosomal cathepsin D activity in lysosomes by 79% in the cortical neurons treated with Aβ_1–42_ oligomers (Fig 6B). There is no significant reduction of cytosolic cathepsin D activity upon treatment with Aβ_1–42_ oligomers in the cytosolic fraction (Fig 6B). The reduction of cathepsin D activity was further examined by immunoblot analysis with cytoplasmic and lysosome-enriched fractions. This reveals decreased levels of cathepsin D protein expression in the lysosome when Aβ_1–42_ oligomers are treated, whereas the ectopic expression of GCase restored cathepsin D protein levels to the levels comparable with the group untreated with Aβ_1–42_ oligomers. Additionally, cytoplasmic cathepsin D levels were examined. No significant difference is found among the different groups (Fig 6C). These results indicate that GCase expression rescues Aβ_1–42_ oligomer-induced lysosomal impairment including the decreased number and size of lysosomes, impaired acidification of cellular compartments, and reduced lysosomal cathepsin D activity.

## Discussion

Despite the growing evidence implicating GCase in neurodegenerative disorders such as PD, DLB, and GD, very little research has explored the relationship of GCase protein levels and enzyme activity to AD pathology except for the finding from Xu and colleagues suggesting that aggregates of Aβ and amyloid precursor protein (APP) are increased in GD mice, raising the possibility that GCase deficiency contributes to AD [41]. In this study, we have demonstrated that considerably lowered GCase protein levels and enzyme activity were observed in the hippocampus of sporadic AD patients and following Aβ_1–42_ oligomer treatment of hippocampal and cortical neurons suggesting that the deficiency of GCase may play a role in AD progression. Importantly, overexpression of GCase yields beneficial protective effects against Aβ_1–42_ oligomer-induced neurotoxicity through facilitating Aβ_1–42_ oligomer degradation by restoring lysosomal activity. It will be interesting to determine whether GCase protein levels and enzyme activity are altered similarly in other brain regions affected by AD and whether diminished GCase levels inversely correlate with Aβ accumulation. Also of interest is the development of AD animal models with increased wild type GCase enzyme activity, perhaps through *GBA1* gene therapy or pharmacological chaperones. Such a system could potentially determine if the same neuroprotective effects observed in this work could be replicated in an animal system. Further studies like these will enhance our understanding of the GCase-associated lysosomal pathways involved in AD.

A number of possible mechanisms have been suggested for Aβ_1-42_-induced neurotoxicity, but the exact mechanism by which Aβ_1–42_ leads to synaptic dysfunction and neuronal death is still controversial. One possible explanation is that lysosomal membrane permeabilization (LMP) may be involved in Αβ neurotoxicity. Neurons treated with Aβ_1–42_ results in neuronal toxicity accompanied by a loss of lysosomal acidification and membrane integrity, which are indicators of LMP [40, 42, 43]. Consistent with prior studies, we confirmed that Aβ_1–42_ oligomer treatment in primary neurons leads to increased intracellular acidification, alterations in lysosome number and size, and impaired cathepsin D activity, suggesting that LMP may be involved in Αβ toxicity. Importantly, the expression of GCase restores the Aβ_1–42_ oligomer-induced LMP, implying that GCase deficiency is required for Aβ_1–42_ oligomer-induced LMP. Many pathogenic factors in neurodegenerative disorders could contribute to LMP [40, 44]. Among them, normal membrane lipid composition is necessary for lysosomal stability, whereas an imbalance in lipid levels promotes LMP [44, 45]. GCase deficiency may contribute to changes in lipid membrane composition by hampering the hydrolysis of glucosylceramide and glucosylsphingosine, which are substrates for GCase and a major source of lipids. Further studies are required to elucidate the exact mechanism by which GCase regulates LMP.

While not explored, there may be another possible mechanism in which GCase regulates Aβ_1–42_ oligomer levels. Interestingly, there is still a reduction in Aβ_1–42_ oligomer levels in neurons expressing GCase with the treatment of lysosomal inhibitors (Fig 3A). This suggests that GCase promotes degradation of Aβ_1–42_ oligomers not only through autophagy-lysosome pathway but also through other protein turnover regulation pathways, such as ubiquitin-proteasome pathway (UPS) [46] and transport mechanisms including endocytosis and exocytosis [47]. Further studies are needed to elucidate whether GCase facilitates UPS to degrade Aβ_1–42_ oligomers or mediates endocytosis and exocytosis of Aβ_1–42_ oligomers.

While our data indicate that GCase protein levels were decreased in brains with AD or Aβ_1–42_ oligomer-treated AD hippocampal or cortical neurons, there is no evidence elucidating the mechanism by which the GCase protein levels are regulated. One possible scenario is that Aβ_1–42_ oligomers could regulate GCase protein levels through reducing the mRNA levels via an unknown transcription factor. Since *GBA1* mRNA are controlled by transcription factor EB (TFEB) [18, 48], it is possible that Aβ_1–42_ oligomers could prevent TFEB nucleus localization thereby reducing *GBA1* mRNA transcription and thus decreasing GCase protein levels. However, it is unlikely that the reduction in GCase protein levels is due to a transcriptional effect since we found no difference in the levels of *GBA1* mRNA in AD hippocampus versus the controls. On the other hand, another possibility for the reduced GCase protein levels is impairment in the quality control of the UPS in AD brains and Aβ_1–42_ oligomer-treated primary neurons. It has recently been demonstrated that numerous E3 ligases, such as parkin, Itch, and c-Abl, regulate the stability of mutant *GBA1*, despite the fact wild-type *GBA1* is not subject to an interaction with the latter E3 ligases [49–51]. The Hsp-90/Hop/cdc39/hsp-27 complex and ERdj3 are also involved in controlling mutant *GBA1* stability via the endoplasmic reticulum-associated degradation (ERAD) system [52–54]. The identification of the E3 ligase involved in the ubiquitin proteasome degradation pathway for wild-type GCase proteins awaits investigation.

It has been suggested that cell-to-cell spreading of amyloid proteins is associated with the pathological progression of various neurodegenerative disorders, including PD and AD [1, 25, 55]. Lysosomal activity is required for degradation of internalized amyloid proteins, and its activity is an important parameter in determining the rate of cell-to-cell amyloid protein spreading [56]. Lee *et al*., has demonstrated that lysosomal dysfunction due to the GCase deficiency facilitates cell-to-cell spreading of α-synuclein aggregates and PD pathological propagation [25]. This leads us to speculate that lysosomal impairment due to diminished GCase expression and enzyme activity as shown in AD could also affect cell-to-cell transmission of Aβ aggregates and the pathoprogression of AD [57]. It would be interesting to determine if GCase plays a role in the propagation of Aβ aggregates in sporadic AD.

In this study, we demonstrate that GCase expression and enzyme activity in the brain of AD patients is lowered and that this deficiency could play a role in the development of AD by inducing lysosomal dysfunction. In addition, we demonstrate that GCase facilitates the clearance of Aβ_1–42_ oligomers and protects against Aβ_1–42_ oligomer-induced neuronal cell death by enhancing lysosomal function. Altogether, our findings indicate that enhancing lysosomal function via GCase could be a disease-modifying therapy and that restoring GCase enzyme activity through pharmacological chaperones might inhibit the pathoprogression of AD.

## Supporting Information

S1 Fig β-galactosidase activity and GCase glycosylation in sporadic AD brain. (A) β-galactosidase activity was measured using lysosome-enriched fractions prepared from the control or AD hippocampus (each group, n = 6). The β-galactosidase activity was quantified and normalized to the control. (Student’s t test, *P < 0.05) (B) The glycosylation state of GCase in the AD and control hippocampus was analyzed by immunoblotting with Endo H treatment.
(TIF)

S2 Fig Overexpression of GCase restores impairment of lysosomal biogenesis induced by Aβ_1–42_ oligomers in neurons. (A) Primary mouse cortical neurons were infected with lenti-control (LV-Cont, n = 3) or lenti-GCase (LV-GC, n = 3) virus at 7 days *in vitro*. After treatment with PBS or 1 μM oligomeric Aβ_1–42_ for 24 h at 10 days *in vitro*, the neurons were immunostained with anti-MAP2 (neuronal marker, purple) and anti-LAMP1 antibodies (lysosomal marker, red).
(TIF)

S1 Table Human post-mortem tissues used in Fig 1 and S1B Fig. Abbreviations: AD, Alzheimer’s disease; PMD, post-mortem delay; N/A, not available. Case# 5, 12, 2282, and 2417 were used for the Endo-H resistance assay in S1B Fig.
(PDF)

## Abbreviations

AD: Alzheimer’s Disease
DLB: Dementia with Lewy Bodies
TDP43: TAR DNA-binding protein 43
GD: Gaucher’s disease
GCase: Glucocerebrosidase
PD: Parkinson’s Disease
TFEB: transcription factor EB
UPS: ubiquitin proteasome system
4-MU: 4-Methylumbelliferyl β-glucophyranoside
DAPI: 4',6-diamidino-2-phenylindole
TUNEL: Terminal deoxynucleotydyl tranferase dUTP nick and labeling
LDH: lactate dehydrogenase
CD: cathepsin
LAMP1: lysosomal-associated membrane protein 1
LMP: lysosomal membrane permeabilization
APP: amyloid precursor protein
E-64d: (2S,3S)-trans-Epoxysuccinyl-L-leucylamido-3-methylbutane ethyl ester
PepA: pepstatin A
Endo H: endoglycosidase H
CBE: conduritol B epoxide
MOI: multiplicity of infection
