# Supplementary figures and images for: Lysosomal Enzyme Glucocerebrosidase Protects against Aβ1-42 Oligomer-Induced Neurotoxicity
Source: PLoS One. 2015 Dec 2;10(12):e0143854. doi: 10.1371/journal.pone.0143854 (PMC4668030; doi:10.1371/journal.pone.0143854)

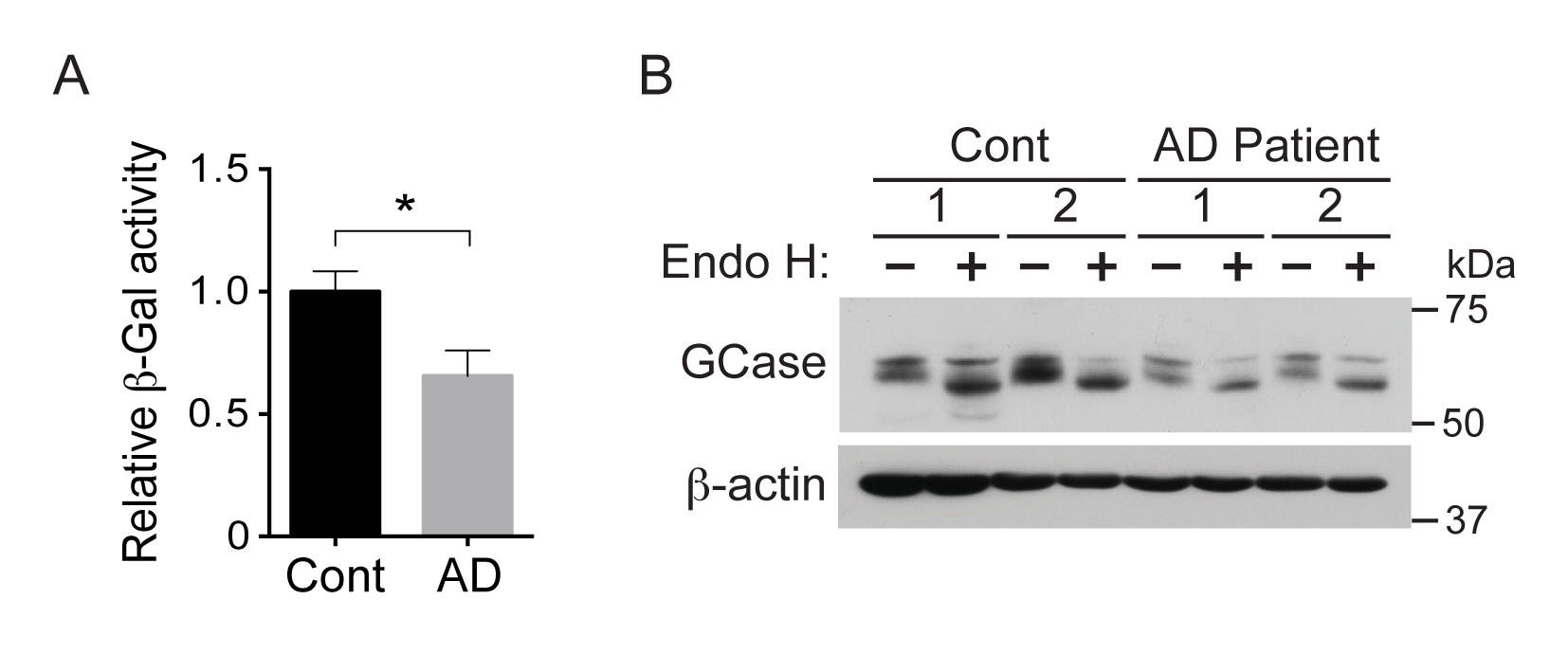

Supplement: S1 Fig — (A) β-galactosidase activity was measured using lysosome-enriched fractions prepared from the control or AD hippocampus (each group, n = 6). The β-galactosidase activity was quantified and normalized to the control. (Student’s t test, *P < 0.05) (B) The glycosylation state of GCase in the AD and control hippocampus was analyzed by immunoblotting with Endo H treatment. (TIF) [file pone.0143854.s001.tif]

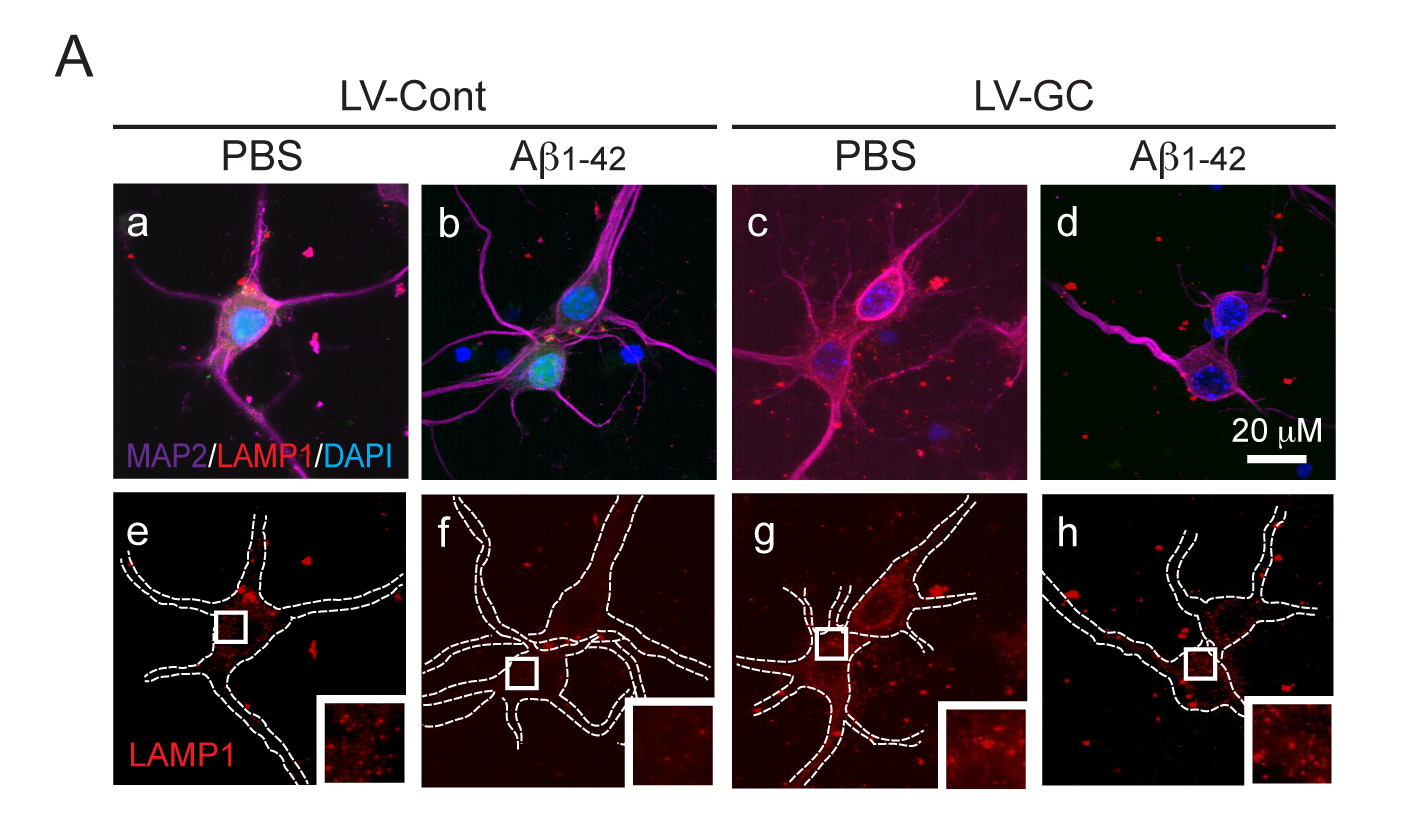

Supplement: S2 Fig — (A) Primary mouse cortical neurons were infected with lenti-control (LV-Cont, n = 3) or lenti-GCase (LV-GC, n = 3) virus at 7 days in vitro. After treatment with PBS or 1 μM oligomeric Aβ1–42 for 24 h at 10 days in vitro, the neurons were immunostained with anti-MAP2 (neuronal marker, purple) and anti-LAMP1 antibodies (lysosomal marker, red). (TIF) [file pone.0143854.s002.tif]
