# Supplementary material for: Lysosomal Enzyme Glucocerebrosidase Protects against Aβ1-42 Oligomer-Induced Neurotoxicity
Source: PLoS One. 2015 Dec 2;10(12):e0143854. doi: 10.1371/journal.pone.0143854 (PMC4668030; doi:10.1371/journal.pone.0143854)
Supplement: S1 Table — Abbreviations: AD, Alzheimer’s disease; PMD, post-mortem delay; N/A, not available. Case# 5, 12, 2282, and 2417 were used for the Endo-H resistance assay in S1B Fig. (PDF) [file pone.0143854.s003.pdf]

S1 Table. Human post-mortem tissues used in Fig. 1 and S1B. Fig.

Case#	Samples	AGE	SEX	Race	PMD (Hr)	Tissue	BRAAK stage
4	Control	63	F	W	22	Hippocampus	
5	Control	69	F	W	13	Hippocampus	
10	Control	67	M	B	23	Hippocampus	
12	Control	64	M	W	11	Hippocampus	
13	Control	59	M	B	20	Hippocampus	
40	Control	72	N/A	N/A	N/A	Hippocampus	
2282	AD	79	F	W	18	Hippocampus	6
2417	AD	61	F	W	5	Hippocampus	6
2430	AD	62	M	N/A	13	Hippocampus	6
2447	AD	65	M	W	11	Hippocampus	6
2454	AD	62	F	W	18	Hippocampus	6
2464	AD	52	M	W	5	Hippocampus	6

Abbreviations: AD, Alzheimer’s disease; PMD, post-mortem delay; N/A, not available

Case# 5, 12, 2282, and 2417 were used for the Endo-H resistance assay in S1B. Fig.
